# Genomic, Morphological and Functional Characterization of Virulent Bacteriophage IME-JL8 Targeting *Citrobacter freundii*

**DOI:** 10.3389/fmicb.2020.585261

**Published:** 2020-11-19

**Authors:** Kaixiang Jia, Nuo Yang, Xiuwen Zhang, Ruopeng Cai, Yang Zhang, Jiaxin Tian, Sayed Haidar Abbas Raza, Yuanhuan Kang, Aidong Qian, Ying Li, Wuwen Sun, Jinyu Shen, Jiayun Yao, Xiaofeng Shan, Lei Zhang, Guiqin Wang

**Affiliations:** ^1^College of Animal Science and Technology, Key Laboratory of Animal Production and Product Quality Safety of Ministry of Education, Jilin Agricultural University, Changchun, China; ^2^Department of Pediatric Neurology, The First Hospital of Jilin University, Changchun, China; ^3^Research Management Office, Jilin Academy of Agricultural Sciences, Changchun, China; ^4^College of Animal Science and Technology, Northwest A&F University, Xianyang, China; ^5^Zhejiang Institute of Freshwater Fisheries, Huzhou, China

**Keywords:** *Citrobacter freundii*, bacteriophage, phage therapy, biofilm, phage genome

## Abstract

*Citrobacter freundii* refers to a fish pathogen extensively reported to be able to cause injury and high mortality. Phage therapy is considered a process to alternatively control bacterial infections and contaminations. In the present study, the isolation of a virulent bacteriophage IME-JL8 isolated from sewage was presented, and such bacteriophage was characterized to be able to infect *Citrobacter freundii* specifically. Phage IME-JL8 has been classified as the member of the *Siphoviridae* family, which exhibits the latent period of 30–40 min. The pH and thermal stability of phage IME-JL8 demonstrated that this bacteriophage achieved a pH range of 4–10 as well as a temperature range of 4, 25, and 37°C. As revealed from the results of whole genomic sequence analysis, IME-JL8 covers a double-stranded genome of 49,838 bp (exhibiting 47.96% G+C content), with 80 putative coding sequences contained. No bacterial virulence- or lysogenesis-related ORF was identified in the IME-JL8 genome, so it could be applicable to phage therapy. As indicated by the *in vitro* experiments, phage IME-JL8 is capable of effectively removing bacteria (the colony count decreased by 6.8 log units at 20 min), and biofilm can be formed in 24 h. According to the *in vivo* experiments, administrating IME-JL8 (1 × 10^7^ PFU) was demonstrated to effectively protect the fish exhibiting a double median lethal dose (2 × 10^9^ CFU/carp). Moreover, the phage treatment led to the decline of pro-inflammatory cytokines in carp with lethal infections. IME-JL8 was reported to induce efficient lysis of *Citrobacter freundii* both *in vitro* and *in vivo*, thereby demonstrating its potential as an alternative treatment strategy for infections attributed to *Citrobacter freundii.*

## Introduction

*Citrobacter freundii* (*C. freundii*) refers to a facultative anaerobic Gram-negative bacillus that has been reported to exist in soil, water, food and the natural environment (e.g., hospitals). It pertains to the family *Enterobacteriaceae*. *C. freundii* belongs to a normal flora of the fish and human intestine, as well as a conditionally pathogenic bacteria capable of infecting people that exhibit low immunity, which will cause several diseases (e.g., pneumonia, meningitis, sepsis, bacteremia, and urinary tract infections) (Anderson et al., 2018; Ando et al., 2018; Liu et al., 2018). Furthermore, *C. freundii* has been associated with numerous diseases and symptoms (e.g., hemorrhagic septicemia, severe enteritis and serious lesions in kidney and gills of catfish; systemic infection in common carp; tail necrosis, septicemia, hemorrhage, as well as reddening of the body in Mozambique tilapia; high mortality in Nile tilapia; cutaneous hemorrhages in zebrafish; tail necrosis, septicemia, hemorrhage as well as reddening of the body in Mozambique tilapia; high mortality in Nile tilapia; gastroenteritis and progressive high-mortality in rainbow trout; systemic infection in common carp) (Zurfluh et al., 2017; Castanheira et al., 2018; Hassen et al., 2020). *C. freundii* has been reported to resist numerous commonly used antibiotics since its discovery. Over the past few years, as impacted by the indiscriminate use of antibiotics and weak supervision of drug-resistant bacteria, a growing number of multi-drug resistant *C. freundii* were clinically detected (Ahad et al., 2017). *C. freundii* can form surface-related complex communities, which was considered biofilms in both food matrices and hospital settings. However, it can resist dehydration, UV radiation, common chemical sanitizers, as well as detergents. As a matter of fact, antimicrobial agents used routinely can inhibit its growth only, while no such treatments have been found effective to remove *C. freundii* in hospital environments (Santos et al., 2017). Phage treatment was described as a promising approach to regulate pathogens and reduce biofilms.

Bacteriophage or phage refer to a type of virus capable of infecting bacteria. Virulent bacteriophages can cause bacteria to lyse and die. Bacteriophages are reported as the most diverse organisms in the biosphere. Unlike the broad-spectrum antibiotics, bacteriophage specific bactericidal function has little effect on the normal flora in the body (Rozand and Feng, 2009; Oliveira et al., 2016). Moreover, phages are capable of penetrating the inner layers of the biofilms and infecting dormant cells, considered to be a significant advantage of phages over antibiotics in killing biofilms. Phage therapy consists of the use of a single phage preparation, as well as a cocktail of multiple phage (Zhang et al., 2018). Since bacteriophage therapy exhibits high efficiency in treating bacterial infections, phages have been extensively applied as antibacterial agents in food production and aquaculture industry.

Bacteriophages lytic against other types of bacteria (e.g., *Escherichia coli* (Sadekuzzaman et al., 2017; Scanlan et al., 2017; Gilcrease and Casjens, 2018), *Pseudomonas aeruginosa* (Tsao et al., 2018; Bru et al., 2019) and *Streptococcus* (Leprohon et al., 2015; Lavelle et al., 2018; Szymczak et al., 2019) have been suggested to effectively disrupt mono-biofilms formed by their respective hosts. In this study, the morphological characterization and full genomic of a novel lytic bacteriophage, IME-JL8, were presented. According to the sequencing and analysis of the IME-JL8 genome, the lack of bacterial virulence or lysogenesis-related ORFs was revealed, so this bacteriophage was proven eligible for use in phage therapy. Besides, the phage can disrupt existing *C. freundii* biofilms. Furthermore, as suggested from the positive results of this study, phage treatment may act as an effective approach and exhibit high potential to prevent and treat *C. freundii*-related disease.

## Materials and Methods

### Animal Feeding

In this study, specific pathogen-free and clinically healthy common carp (average weight 50 ± 1 g) specimens were provided by a commercial fish farm and employed for subsequent studies. Fish were maintained in 200 L flow-through tanks at 25 ± 1°C under natural photoperiod. Fish were fed with commercial diet twice a day at a feeding rate of 1% body weight.

### Bacterial Strains and Phages

Table 1 lists the bacterial strains used in the present study. The antibiotic sensitivity test on *C. freundii* CF8 was showed in Supplementary Table S1. The *C. freundii* strains were incubated in 30% glycerol at −80°C and subsequently cultured in Brain-Heart Infusion (BHI) broth at 37°C.

**TABLE 1:** Bactericidal spectrum of IME- JL8.

Bacterial strain	Date of collection (Year-Month)	Efficiency of plating of IME- JL8
*Citrobacter freundii* 2052^2^	_^5^	N
*Citrobacter freundii* 2262^2^	_^5^	N
*Citrobacter freundii* 19^2^	_^5^	N
*Citrobacter freundii* 78^2^	_^5^	N
*Citrobacter freundii* 2151^2^	_^5^	N
*Citrobacter freundii* 1152^2^	_^5^	N
*Citrobacter freundii* 15^2^	_^5^	N
*Citrobacter freundii* 1136^2^	_^5^	N
*Citrobacter freundii* 77^2^	_^5^	N
*Citrobacter freundii* 1025^2^	_^5^	N
*Citrobacter freundii* 223^2^	_^5^	N
*Citrobacter freundii* 1864^2^	_^5^	N
*Citrobacter freundii* CF8^1^	2016–2011	Y
*Citrobacter freundii* CF2^1^	2016–2011	N
*Citrobacter freundii* CF3^1^	2016–2011	N
*Escherichia coli* ATCC 25922^3^	_^5^	N
*Staphylococcus aureus* ATCC 25923^3^	_^5^	N
*Bacillus subtilis* ATCC14579^3^	_^5^	N
*Salmonella sp.* ATCC10248^3^	_^5^	N
*Klebsiella pneumoniae* BAA-2146^3^	_^5^	N
*Streptococcus sp.* CVCC606^4^	_^5^	N

*^1^iIsolated from the carp kept in our lab (Changchun, Jilin province, China). ^2^Given by professer Yigang Tong. ^3^Purchased from American Type Culture Collection (ATCC). ^4^Purchased from China Institute of Veterinary Drug Control (CVCC). ^5^No data are available. ^6^Y, yes; N, No.*

In the present study, *C. freundii* CF8 was adopted for phage propagation. Sewage samples of 500 mL were harvested from the sewerage systems in Changchun, China. The bacteriophage was counted and purified with the double-layer agar plate method. To be specific, the phage was purified via repeated double-layer agar plate till the plaques became homogeneous. Spot tests were performed to identify the presence of phage, and the phages were amplified and incubated at 4 and −80°C in glycerol (3:1 [v/v]).

### Host Range Determination

This study characterized the host range of the phage with the double-layer agar plate method following the previous description with minor modification (Yang et al., 2015). Table 1 elucidates the bacterial strains used in the study.

### Biological Characteristics of the Phage

On the whole, the biological characteristics of phage IME-JL8 consist of the killing curves of the phage under a range of multiplicities of infection (MOI), One-step growth, thermal stability, pH stability, as well as storage stability. To determine phage titer and MOI, the double-layer agar plate method was employed, and plaques were measured after the strains were cultured for 8 h. The maximum MOI refers to the optimal MOI for this bacteriophage (Al-Zubidi et al., 2019).

The one-step growth curve of the phage was determined following the previous description with some modifications (Al-Zubidi et al., 2019). In summary, the *C. freundii* CF8 strain (mid-exponential phase) was cultured, harvested and resuspended in fresh BHI broth. The phage was added at a MOI for 10 min adsorption at 37°C. The phage–host mixture underwent the centrifugation at 6797 × *g* for 10 min at 4°C. The pellets were suspended in 10 mL of fresh BHI medium that was preheated at 37°C. Then, the achieved suspension was incubated at 37°C while being shaken at 140 rpm. Samples were taken at 10 min intervals over 160 min, immediately diluted and subsequently plated for phage titration with a double-agar layer technique.

The susceptibility of phage IME-JL8 to varying pH was determined by incubating the bacteriophage in BHI broth adjusted to pH 4–10 for 1 h, complying with the previous description (Cai et al., 2019). The thermal stability of phage IME-JL8 was determined by incubating IME-JL8 in BHI broth at 4, 25, 37, 50, 60, 70, and 80°C for 80 min. Moreover, to test phage stability after long-term storage, aliquots of phage IME-JL8 suspensions were incubated at 4°C for 1 year. Furthermore, a double layer agar plate was employed in the pH stability, thermal stability and storage stability assay.

### Phage Morphology and SDS-PAGE Analysis of Structural Proteins

The concentration and purification of phage were conducted following the previous description with minor modifications (Gong et al., 2016). After the IME-JL8 phage was cultured in a large scale, BHI lysates underwent the centrifugation at 6797 × *g* for 10 min at 4°C to remove cell debris. In the presence of 10% polyethylene glycol 8000 and 1 M NaCl, phage particles were precipitated from culture supernatant and subsequently dissolved in 5 mL PBS. The phage suspension was placed on the top of a discontinuous CsCl gradient (1.45, 1.50, 1.70 g/mL) and underwent centrifugation at 126,100 × *g* for 3 h at 4°C. The phage band was harvested and dialyzed. Next, a sample was applied to copper grids stained with phosphotungstic acid (PTA, 2% w/v) negatively and characterized under a transmission electron microscopy (TEM) (JEOL JEM-1200EXII, Japan Electronics and Optics Laboratory, Tokyo, Japan) at an accelerating voltage of 80 kV.

The sodium dodecyl sulfate-polyacrylamide gel electrophoresis (SDS-PAGE) was performed to define the major proteins in bacteriophages IME-JL8. The concentrated phage particles were mixed with the sample buffer (supplemented by 2 mM 2-mercaptoethanol), and the samples were heated at 95°C for 7 min. Afterwards, 10 μL of lysate was loaded directly onto standard 15% SDS-PAGE gels for protein separation. Next, the gels were stained with Instant Blue (Expedeon).

### Phage Genome Sequencing

With the Universal Phage Genomic DNA Extraction Kit (Knogen, Guangzhou, China), genomic phage DNA was isolated from produced high titer phage particles (≥10^10^ PFU/mL) and incubated at −20°C for sequencing. Based on Illumina HiSeq 2500 sequencing, the whole genome sequencing of phage IME-JL8 was performed by Suzhou GENE-WIZ Biotechnology Co., Ltd. The genome sequences were assembled with the SOAP denovo package. With CGView^1^, the circle map of the IME-JL8 genome was generated. Genome annotation and potential open reading frames (ORFs) were assessed and analyzed with BLAST and GeneMarkS. Next, global genome comparisons were drawn with Mauve (version 2.3.1).

### Biofilm Assay

Based on a 96-well microtiter plate method presented in one existing study (Zhang et al., 2018) with minor modifications, the ability of *C. freundii* CF8 strain to form biofilms was assessed. In brief, the CF8 strain was inoculated into 5 mL of sterile TSB medium and grown for 16 h at 37°C. A 1:100 dilution of culture was transferred into fresh BHI medium, and 200 μL (10^6^ CFU/mL) of the culture was introduced to the wells in untreated 96-well microtiter plates. The wells supplemented by only BHI medium acted as the negative controls. The plates were incubated for 24 h at 37°C without being shaken. The medium was renewed per 12 h and then underwent the phage treatment at a final titer of 7 log10 PFU. MIC sensitive antibiotics cefoperazone sodium, tetracycline and phosphate-buffered saline (PBS) were used as the control group under the same conditions (Akturk et al., 2019). The ability of different concentrations of cefoperazone sodium and tetracycline to remove biofilms was measured. Samples received the further incubation at for 6 h 30°C. After different group treatment, each well was washed with PBS for three times and then air-dried. After the well was washed with PBS, 98% methanol was added and left for 10 min. Then the methanol was removed, and the plates were air-dried again. Overall, the samples were stained with 1% crystal violet solution for 45 min followed by elution with 33% acetic acid. The OD value sample was identified by a spectrometer at a wavelength of 590 nm.

In the SEM assay, the *C. freundii* CF8 strain was cultured in 5 mL of BHI to an OD_600_ of 0.6 at 37°C while being shaken at 180 rpm. The cultures were centrifuged and resuspended in BHI medium exhibiting equal volumes. Subsequently, 200 μL of bacterial culture and 200 μL of fresh BHI were added to each well in a 24-well microtiter plate; the wells were covered with sterile 14-mm-diameter glass sheets that were preadministrated with polylysine. Next, the 24-well microtiter plate underwent the incubation without being shaken 24 h at 37°C. After incubation for 24 h, the non-adherent cells were removed, and the wells were washed three times with sterile PBS. The biofilms were administrated with 7 log10 PFU of phage IME-JL8 diluted in buffer for 6 h at 37°C. PBS acted as a negative control. The biofilm lysates were immobilized with 5% glutaraldehyde, dehydrated under a range of concentrations (i.e., 20, 50, 70, 90, and 100%) of ethanol, and then freeze-dried before the scanning electron microscopy (SEM) was used for characterization (Hitachi S-3400N; Hitachi High-Technologies Europe GmbH, Krefeld, Germany).

### Antimicrobial Activity of the Phage IME-JL8

For *in vitro* lysis assays, the *C. freundii* CF8 strain was cultured to log phase in BHI broth (OD_600_ = 0.6) and cleaned three times with PBS. Next, the produced IME-JL8 dilutions in PBS buffer were inoculated into double-strength BHI. The mixture was diluted to normal strength BHI and then normalized to a final bacterial count of 10^8^ CFU/mL; meantime, phage was added at a MOI of 0.01 in the tubes. The cell viability for CFU/ml following exposure was measured at 10, 20, 30, 40, 50, and 60 min after incubation. Bacterial growth without phage addition acted as the control. The number of viable CF8 cells (CFU/mL) was ascertained by serial dilution and plated on BHI agar plates.

The therapeutic potential of phage was assessed for its ability to treat the infection of *C. freundii* in fish. First, the carps were randomly split into two groups. Carps in one group were injected intraperitoneally with 200 μL / fish of phage IME-JL8 (1 × 10^8^ PFU/mL), while the other group was the blank control administrated with PBS over a 10-day follow-up. To determine the bacterial dose leading to 100% mortality over a 7-day follow-up (the minimal lethal dose [MLD]), groups of 6 carp per experiment were injected intraperitoneally (i.p.) with different inocula of *C. freundii* CF8 (10^7^, 10^8^, 10^9^, 10^10^, and 10^11^ CFU, 100 μL/fish). The number of dead fish was recorded daily. Once the MLD had been determined, 2 × MLD acted as the infective inoculum (challenge dose).

Following infection with 2 × MLD (2 × 10^9^ CFU/fish, 100 μL/fish) of *C. freundii*, 1 h later, the fish were treated intraperitoneally with IME-JL8 at different concentrations (1 × 10^6^, 1 × 10^7^ or 1 × 10^8^ PFU/mL, 100 μL/fish) (*n* = 6 in respective group) (Gu et al., 2016; Cheng et al., 2017; Zhang et al., 2018). To further test the bactericidal ability of bacteriophages *in vivo*, the fish were infected with 2 × MLD (2 × 10^9^ CFU/fish, 100 μL/fish) of *C. freundii*, 12 and 24 h later, the fish were treated intraperitoneally with IME-JL8 at concentrations (1 × 10^8^ PFU/mL, 100 μL/fish) (*n* = 6 in respective group). The control group was administrated with an equal amount of PBS (100 μL/fish) buffer under the identical conditions. The survival rate was recorded per day for 7 days.

The concentrations of the cytokines (TNF-α, IFN-γ, and IL-1β) in the blood samples in different groups at 1, 6, 12, or 24 h after being challenged were quantified with qPCR based on the methods of existing studies. All qPCRs samples were performed with three replicates. The primers for the immune-related genes studied and β-actin, as examined as a housekeeping gene (Table 2).

**TABLE 2:** Sequences and conditions of the primers used in RT-PCR analysis.

Gene	Sequence(5′-3′)	Accession	PCR product (bp)	Cycling conditions	No. of cycles
IL-1β	F:AACTGATGACCCGAATGGAAC R:CACCTTCTCCCAGTCGTCAAA	AB010701	133	95°C–30 s 61°C–30 s	40
IFN-γ	F: AACAGTCGGGTGTCGCAAG R:TCAGCAAACATACTCCCCAG	AB376666	141	95°C–30 s 62°C–30 s	40
TNF-α	F: TTATGTCGGTGCGGCCTTC R: AGGTCTTTCCGTTGTCGCTTT	AJ311800.2	101	95°C–30 s 63°C–30 s	40
β-actin	F: CAAGATGATGGTGTGCCAAGTG R:TCTGTCTCCGGCACGAAGTA	M24113.1	352	95°C–30 s 62°C–30 s	40

### Data Analysis

SPSS version 13.0 (SPSS, Inc., Chicago, IL, United States) was employed to statistically analyze other experimental data with a one-way analysis of variance (ANOVA). GraphPad Prism 5 (GraphPad Software, Inc., La Jolla, CA, United States). A *p-value* < 0.05 was considered to exhibit statistical significance. Error bars represent standard deviation of the mean.

## Results

### Phage Purification and Characteristics

**FIGURE 1:**
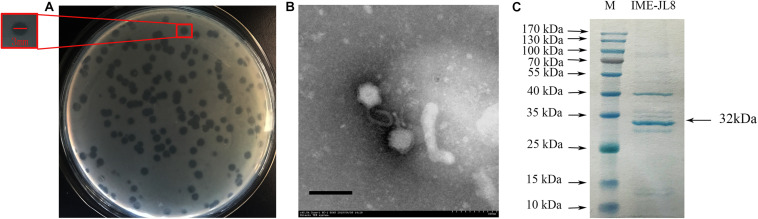
Phage morphology and structural protein detection. **(A)** Bacteriophage IME-JL8 spotted onto *C. freundii* CF8 culture on BHI agar. Each single plaque was ≈3 mm diameter. **(B)** TEM image of IME-JL8 revealing *Siphoviridae* bacteriophage with long tail and icosahedral head. **(C)** SDS-polyacrylamide gel (15%) electrophoresis of IME-JL8 structural proteins. M: molecular mass marker. Lane 1, IME-JL8 proteins. The most abundant structural protein was about 32 kDa.

**FIGURE 2:**
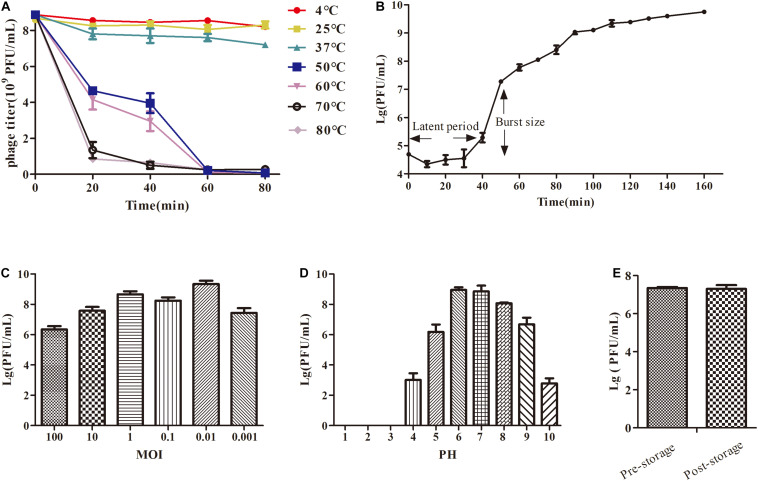
The growth characteristics and stability tests of IME-JL8. **(A)** Thermal stability: phage particles were incubated at different temperatures as indicated. **(B)** A one-step growth curve of IME-JL8. **(C)** Titers of the phage under different MOI (phage/bacteria = 0.001, 0.01, 0.1, 1, 10, and 100), as represented in the *Y*-axis. At the MOI of 0. 01, IME-JL8 peaked the maximum titers. **(D)** pH stability: IME-JL8 were incubated under different pH values. **(E)** Long-term storage stability: the titer of pre-storage and post-storage (for 1 year) of IME-JL8.

Based on plaque purification, *C. freundii* phage IME-JL8 was isolated from the Chang Chun sewage. As incubated with *C. freundii* CF8, IME-JL8 formed clear plaques (2–3 mm diameter) on lawns of CF8 (Figure 1A). Apart from CF8, IME-JL8 failed to lyse other *C. freundii* strains and other species (Table 1). The maximum titers of IME-JL8 could reach 10^9^ PFU/mL under the MOI of 0.01 (Figure 2C). Thus, the one-step growth curve of IME-JL8 propagated on the CF8 strain in BHI broth was plotted at a MOI of 0.01. Figure 2B suggests that the mentioned one-step growth curve revealed that the latent and rise periods were approximately 30 and 40 min.

Phage IME-JL8 can basically maintain its original activity between 4, 25, and 37 °C (Figure 2A). Simultaneously, IME-JL8 can maintain high activity at pH 4–10 (Figure 2D). Under pH below 4 or above 10, the phage activity declined sharply. Furthermore, the titer of IME-JL8 almost remained unchanged after 1 year of storage at 4°C (Figure 2E).

From the structural perspective, the transmission electron microscopy revealed that IME-JL8 exhibited morphological characteristics of the family *Siphoviridae*, with a long flexible tail and an icosahedral head. The diameter of the isometric head nearly reached 68 nm (Figure 1B). To characterize the IME-JL8 phage in depth, the structural protein composition was analyzed by SDS-PAGE. Following gel electrophoresis, 11 major protein bands were visualized by Coomassie staining (Figure 1C). The size of the most abundant major structural protein reached about 32 kDa.

### Overview of the Phage IME-JL8 Genome

**FIGURE 3:**
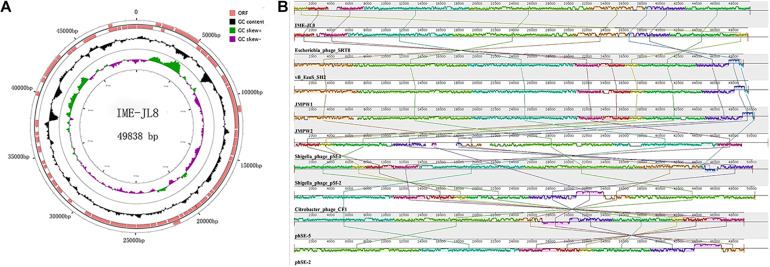
Genome characteristics of IME-JL8. **(A)** The genetic and physical organization of the IME-JL8 genome. ORFs with 80 or more residues are represented by arrows and the arrowheads point in the transcription direction. The figure also maps the G+C content, which is skewed. **(B)** Multiple genome alignments among IME-JL8 and other homologous phages. Similarity is indicated by the height of the bars, complying with the average level of conservation in that region of the genome sequence. The homologous phages are marked in the picture.

To elucidate this phage at the genetic level, the complete genome sequence of IME-JL8 was sequenced, analyzed and then deposited in GenBank under accession number (MT 023084). The IME-JL8 phage genome was a 49,838 bp contiguous sequence of double-stranded DNA, linear, and the overall G + C content was 47.96%. The complete genome of IME-JL8 encoded 80 assessed open reading frames (ORFs), and the arrangement of the mentioned putative ORFs was mapped at the whole-genome level (Figure 3A). The gene-coding potential of the global genome was 92.64%, with an average ORF size of 539.45 bp. Among these ORFs, 32 (40 %) of the initiation codons were ATG, 27 (33.75%) of the initiation codons were TTG, 13 (16.25 %) starting codons were GTG, and 8 (10 %) was GCG (Figure 3A).

All assessed proteins were examined for similarity to known bacterial and phage sequences deposited in the public National Center for Biotechnology Information (NCBI) databases. The modular organization of the IME-JL8 genome displayed four major conserved patterns, i.e., gene expression, gene synthesis, host lysis and virion assembly. The maximum ORF of IME-JL8 was ORF 9 (33.12899 kDa), adjacent to the structure proteins and exhibiting 99% identity with phage SRT8 (putative ATP-dependent helicase). Moreover, there were two other ORFs (i.e., ORF68 and ORF69), encoding a putative endolysin and holin protein. However, no ORFs were associated with drug resistance or lysogeny (e.g., site-specific integrases and repressors). As revealed from bioinformatics annotation and analysis, no similarities were identified between genes or proteins encoded by IME-JL8 and genes or proteins for other factors known to impact virulence during acute or chronic infection by *C. freundii.*

The full-length genome of IME-JL8 was blasted and then analyzed with the genes in the GenBank database. As revealed from a comparative analysis, the genome was highly similar to that of the *Escherichia* phage SRT8, vB_EcoS_SH2, JMPW1, JMPW2, *Shigella* phage pSf-2, *Citrobacter* phage CF1, *Salmonella* phage phSE-5, phSE-2 (Figure 3B). According to nucleotide blast, the genome does not exhibit 100% identical sequence homologies to currently known phage strains in GenBank, suggesting that it is a novel phage strain.

### The Bactericidal Effect of the Phage IME-JL8 *in vitro*

**FIGURE 4:**
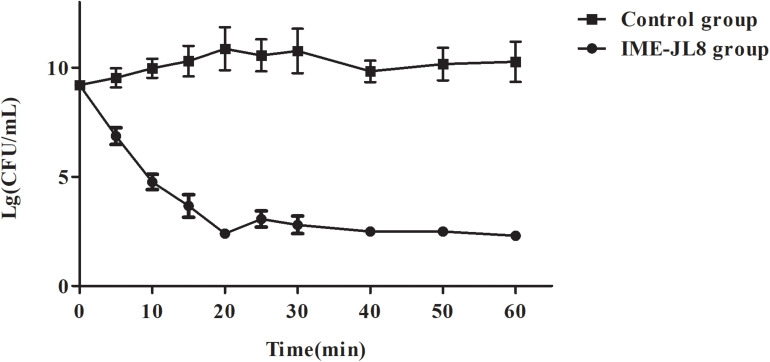
The bactericidal activity of IME-JL8 to *C. freundii* CF8. The CFU/mL decrease of the *C. freundii* CF8 culture was adopted to assess the bactericidal activity of IME-JL8. A final bacterial (*C. freundii* CF8) count of 10^8^ CFU/mL and phage IME-JL8 was added at a MOI of 0.01 in the tubes. The bacterial administrated with PBS as control. Each data is expressed as mean ± SD from three biological experiments.

To experimentally determine the bactericidal activity of IME-JL8 *in vitro*, the time killing assay was performed. As demonstrated from the results of the lytic assay, when IME-JL8 were incubated with CF8, the colony count decreased by 6.8 log units at 20 min after treatment (Figure 4).

**FIGURE 5:**
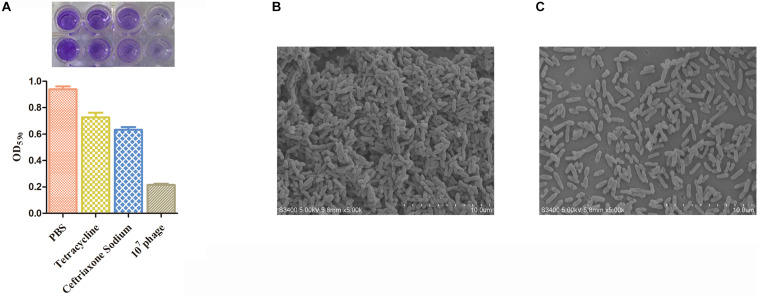
Biofilm removal activity of IME-JL8. The reduction of biofilm after phage treatment with titers of 7 log10 PFU/mL and MIC concentration of cefoperazone sodium and tetracycline for 6 h. **(A)** Biofilm formation state was indicated by OD_590_ values. **(B,C)** Micrographs of biofilms taken under the scanning electron microscopy. **(B)** The SEM of bacteria incubated overnight at 37°C. **(C)** The SEM of bacteria administrated with IME-JL8 for 6 h. The bars represent 10 μm.

The ability of CF8 to form biofilms was experimentally determined. We found that CF8 formed the most stable biofilms at 24 h in the previous result (OD_590_ = 0.374, 6 h; OD_590_ = 0.628, 12 h; OD_590_ = 0.927,24 h; OD_590_ = 0.784, 48 h; OD_590_ = 0.703, 72 h). Figure 5A illustrates the reduction of biofilm after phage treatment with titers of 7 log10 PFU/mL for 6 h. The CF8 biofilm removal activity of 77.7% observed when phage IME-JL8 was applied to a final titre of 7 log10. Two sensitive antibiotics cefoperazone sodium and tetracycline were selected depending on their mechanism of action: cell wall synthesis inhibitor (cefoperazone sodium), protein synthesis inhibitor (tetracycline). When we used MIC cefoperazone sodium and tetracycline as the control group to remove biofilms, the CF8 biofilms removal activities were 30.7 and 20.3%, respectively (Figure 5A). When using 32 × MIC cefoperazone sodium and tetracycline, the CF8 biofilms removal activities were 82.5% (Supplementary Figure S1). Furthermore, micrographs captured under the scanning electron microscopy (SEM) clearly indicated the structurally complex variation, and the number of live cells in the PBS treatment group was found significantly higher than in the phage IME-JL8 treatment group (Figures 5B,C).

### Phage Therapeutic Study

**FIGURE 6:**
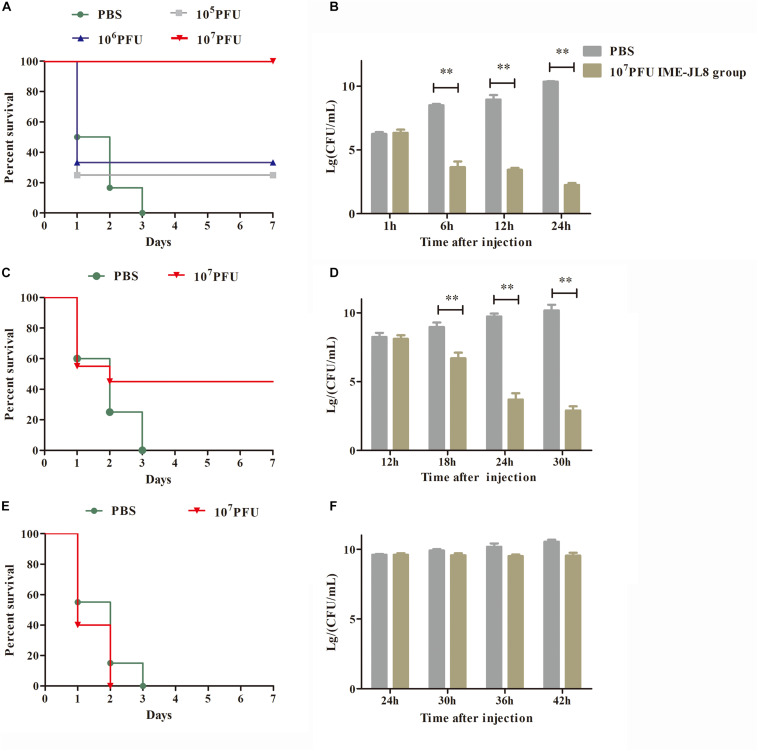
IME-JL8 therapeutic study. The fish were intraperitoneal injection of 2 × minimum lethal dose (MLD) (2 × 10^9^ CFU/carp) of *C. freundii* CF8. **(A–E)** Survival rate of different groups. One hours later, 10^5^, 10^6^, and 10^7^ PFU of IME-JL8 were introduced intraperitoneal **(A)**. After injection of *C. freundii* CF8 (2 × 10^9^ CFU/carp) 12 h later **(C)**, 24 h later **(E)**, 10^7^ PFU phage of IME-JL8 were introduced intraperitoneal. Control fish were administrated with PBS under the identical conditions. **(B,D,F)** Colony counts of bacteria changed in the blood. After injection of *C. freundii* CF8 (2 × 10^9^ CFU/carp) 1 h later **(B)**, 12 h later **(D)**, 24 h later **(F)**. colony counts of bacteria changed in the blood at regular intervals (*n* = 6 in each group). Control fish were administrated with PBS under the identical conditions. The means and standard deviations are represented as points with error bars.

In the safety test, the fish exhibited the survival rate of 100%, demonstrating that IME-JL8 had no side effects on carps. Intraperitoneal injection of 2 × minimum lethal dose (MLD) (2 × 10^9^ CFU/carp) of *C. freundii* CF8 was sufficient to produce a 100% mortality rate within 3 days, in contrast, all the carps administrated with phage IME-JL8 (1 × 10^8^ PFU/mL, 100 μL/carp) recovered (Figure 6A). After infection CF8 (2 × 10^9^ CFU/carp) for 1 h, the bacterial loads reached >10^6^ CFU/mL in the blood (Figure 6B). At this time, the carp were administrated with phage IME-JL8 (1 × 10^8^ PFU/mL, 100 μL/carp). As shown in Figure 6B, the bacteremia greatly decreased reaching approximately 3.3 log units after 12 h in the blood. By contrast, the bacterial loads in the carps administrated with PBS reached approximately 8.6 log units after 12 h in the blood. After infection CF8 (2 × 10^9^ CFU/carp) for 12 h, the bacterial loads reached >10^8^ CFU/mL in the blood (Figure 6D). At this time, the carp were administrated with phage IME-JL8 (1 × 10^8^ PFU/mL, 100 μL/carp). As shown in Figure 6C, phage IME-JL8 was sufficient to produce a 45% survival rate within 3 days. The bacteremia greatly decreased reaching approximately 3.7 log units after 24 h in the blood. By contrast, the bacterial loads in the carps administrated with PBS reached approximately 9.7 log units after 24 h in the blood (Figure 6D). However, for the 24 h post-treatment, the fish died within 2 days in the phage IME-JL8 treated group (Figure 6E) and there was no significant decrease in the bacterial load in the blood (Figure 6F).

**FIGURE 7:**
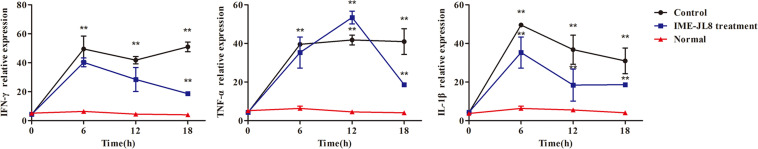
Cytokine levels. The fish were injected intraperitoneally with *C. freundii* CF8 (2 × 10^9^ CFU/carp). One hour later, IME-JL8 (10^8^ PFU/mL, 100 μL/fish) or PBS (100 μL/fish) were introduced intraperitoneally to the fish. At the indicated times, the levels of TNF-α, IL-1β and IFN-γ in the blood of the different treatment groups were determined by qRT-PCR. **P values of <0.05 compared with the PBS treated control. The mentioned experiment was repeated three times. Each data is expressed as mean ± SD from three biological experiments.

By qRT-PCR analysis, the levels of TNF-α, IL-1β and IFN-γ in the blood of the different treatment groups were examined. As shown in Figure 7, the cytokines levels of IL-1β and IFN-γ in the blood at 6 h increased rapidly and then significantly decreased after IME-JL8 treatment. However, the cytokines levels of TNF-α peaked at 12 h and then decreased in the IME-JL8 treatment group. TNF-α, IL-1β, and IFN-γ expressions in the blood exhibited significantly lower levels at 18 h compared with the control groups.

## Discussion

Multidrug-resistant bacteria are becoming increasingly prevalent worldwide. *Citrobacter freundii* refers to a fish pathogen known for its ability to cause injury and high mortality (Yang et al., 2018). For the study, the clinical isolate *C. freundii* CF8 applied in the present study demonstrated a high resistance to antibiotics and was a robust biofilm producer. It is known that multi-resistant and strongly biofilm forming strains have virulence factors, probably contributing to the strain pathogenicity and to the difficulty in treating the disease in fish (Zurfluh et al., 2017). Accordingly, precaution measures are required to manage infections by *C. freundii* strain.

In the present study, our results showed that a newly isolated bacteriophage IME-JL8 can lyse the actively growing cells of *C. freundii* efficiently. Phages can be isolated from a wide variety of sources e.g., sea water, sewage water/sludge ponds etc. (Abedon, 2015; Xu et al., 2015; Jurac et al., 2019). Phage IME-JL8 was isolated from sewage. Sewage is a rich source of phages that infect pathogenic bacteria, e.g., *E. coli, P. aeruginosa*, and *Salmonella*. Thus, it acts as an appropriate sample source for conventional bacterial phage isolation. Physical and chemical stability are critical for phages to be applied for clinical antimicrobial preparations. In the present study, the titers of IME-JL8 exhibited stability in the floating range of conventional pH (5–9) and temperature (<50°C), and the titer of this phage could be long maintained at 4°C. Electron microscopy indicated that IME-JL8 had morphological characteristics of the family *Siphoviridae*, with a long flexible tail and an icosahedral head. This is different from the *C. freundii* phage that has been discovered (Chaudhry et al., 2014; Zhao et al., 2016). The *C. freundii* phage they found were all short tails. Similar to these two phages, IME-JL8 was also highly specific to the bacteria, only sensitive to their host bacteria. Moreover, antibiotic resistance genes or putative virulence factors were not reported in this phage by genomics analysis (Debarbieux et al., 2018; Santos et al., 2018; Straus and Bo, 2018). All the mentioned factors strongly suggest that IME-JL8 could be a potential therapeutic phage against multiple *C. freundii* infections.

Besides biological characteristics, the present study also analyzed the genomic information of IME-JL8. The genome size of IME-JL8 is 49,838 bp, which is less than that of other *C. freundii* phage no. KM236237 (178,171 bp) and no. KT001915 (172,733 bp) (Hwang et al., 2015; LeSage et al., 2015). Similar to these two phages, we did not find putative virulence factors in IME-JL8. Comparative genome analyses revealed the close associations of *Escherichia* phage SRT8, vB_EcoS_SH2, JMPW1, JMPW2, *Shigella* phage pSf-2, *Citrobacter* phage CF1, *Salmonella* phage phSE-5, phSE-2 and suggested molecular clues to elucidate host adaptations in the relevant phages mentioned.

Since bacterial biofilms are highly resistant and resilient to conventional antibacterial therapy, it has been difficult to combat the mentioned infections. An innovative alternative to the bio-control of bacterial biofilms could be the use of bacteriophages, which are specific, non-toxic and self-proliferating, as well as capable of penetrating into biofilms (Kabwe et al., 2019). In this report, the capacity for IME-JL8 to disrupt established *Citrobacter freundii* biofilms was demonstrated. As suggested from the mentioned results, phage therapy on 24-well microplate can effectively reduce the biofilms. It has been reported that treatment with the phage eradicated post-treated biofilm in (44–63%). Crystal violet (CV) was adopted to stain the biofilms; subsequently, the OD_590_ was determined. Phages for *Actinomyces naeslundii*, *Enterococcus faecalis*, *Fusobacterium nucleatum*, *Lactobacillus* spp., *Neisseria* spp., *Streptococcus* spp., and *Veillonella* spp. were isolated and characterized; then, they were reported to effectively reduce the biofilms (Abedon, 2016; Hansen et al., 2019; Issa et al., 2019). In this part, we used two sensitive antibiotics cefoperazone sodium and tetracycline as control group to analyze the effect of antibiotics on bacteria in the biofilm state. The results showed that when we used MIC cefoperazone sodium and tetracycline to remove biofilms, the CF8 biofilms removal activities were 30.7 and 20.3%, respectively (Figure 5A). The phenomenon was observed between the concentrations of cefoperazone sodium and tetracycline on the biofilm killing eradication. The biofilm killing eradication rate increased with the increase of antibiotic concentration. When using 32 × MIC cefoperazone sodium and tetracycline, the CF8 biofilms removal activities was the same as phage-treated group (Supplementary Figure S1). The results were similar to those of previous studies (Akturk et al., 2019). It was necessary to increase gentamicin concentration to obtain a similar killing effect as occurs in the bacteria with biofilms.

To assess the therapeutic effect of IME-JL8, different phage doses were administered intraperitoneally 1 h after the injection. All the carps administrated with phage IME-JL8 (1 × 10^8^ PFU/mL, 100 μL/carp) recovered (Figure 6A). After infection CF8 (2 × 10^9^ CFU/carp) for 1 h, the bacterial loads reached >10^6^ CFU/mL in the blood (Figure 6B). Meantime, the carp were administrated with phage IME-JL8 (1 × 10^8^ PFU/mL, 100 μL/carp). Figure 6B suggested that the bacteremia significantly decreased, reaching approximately 3.3 log units after 12 h in the blood. However, after infection CF8 (2 × 10^9^ CFU/carp) for 12 and 24 h, phage IME-JL8 was sufficient to produce a 45% survival rate within 3 days for 12 h. For the 24 h post-treatment, the fish died within 2 days in the phage IME-JL8 treated group and there was no significant decrease in the bacterial load in the blood. The results showed that phage treatment was effective within 12 h of bacterial infection of fish. The levels of TNF-α, IL-1β, and IFN-γ in the blood of the different treatment groups were determined experimentally by qRT-PCR analysis. The levels of three cytokines increased and peaked at 12 and 6 h, respectively, then decreased. TNF-α, IL-1β, and IFN-γ expressions in the blood exhibited significantly lower levels at 18 h compared with the control groups. The phenomenon is similar to the cytokine secretion of phage X1 (Xue et al., 2020) and phage VB-SavM-JYL01 (Ji et al., 2019) in the treatment of *Yersinia* and *S. aureus*. Some studies have shown that phages have anti-inflammatory properties that reduce the secretion of pro-inflammatory factors. When serum of mice infected with *S. aureus* was treated with phage, the production of TNF-α and IL-6 were reduced, and this phenomenon was also observed in serum samples from some patients treated with phage therapy for bacterial infection (Leung and Weitz, 2017). However, the detailed mechanism of how bacteriophages induce anti-inflammatory effects remains unclear.

During phage therapy, safety is primarily concerned with for therapeutic application on farms or in the food chain. Serious side effects from endotoxin have been rarely reported in the early literature concerning experimental phage therapy. Furthermore, in the present study, the safety of IME-JL8 was determined experimentally before the treatment and prevention trial. The survival rate of the fish reached 100%, demonstrating that lysates of phage are safe and reliable.

Since bacteriophage exhibits rigorous host specificity, and bacteria will develop resistance to bacteriophage in mutual evolution with bacteriophage, single bacteriophage preparation cannot satisfy the treatment requirements. The phage cocktail preparation (cocktail therapy) and personalized phage treatment method may be one of the subsequent research directions of phage treatment (Heyse et al., 2015; Kim et al., 2017; Cieplak et al., 2018). Moreover, some bacteriophages carry resistance genes and even virulence genes, making them unlikely to use directly for treatment. Thus, the isolation, identification, whole genome sequencing and genomic analysis of the bacteriophage are of high significance. It can obtain comprehensive and clear genetic information and evolution information of the bacteriophage, as well as theoretically underpinning bacteriophage therapy.

## Conclusion

In summary we isolate a newly phage IME-JL8 using a *C. freundii* strain originating from the fish as the host. We have identified its biological characteristics as well. The efficient and lytic activity and genomic characteristics of IME-JL8 make it eligible for use in phage therapy. Indeed, IME-JL8 exhibits great therapeutic potential for the treatment in fish caused by *C. freundii*.

## Data Availability Statement

The datasets presented in this study can be found in online repositories. The names of the repository/repositories and accession number(s) can be found in the article/Supplementary Material.

## Author Contributions

LZ: conceptualization. NY and XZ: formal analysis. RC and JT: investigation. YZ and YK: project administration. AQ: resources. YL, WS, JS, XS, and JY: software. GW and LZ: Supervision. KJ: writing – original draft. SR: writing – review and editing. All authors contributed to the article and approved the submitted version.

## Conflict of Interest

The authors declare that the research was conducted in the absence of any commercial or financial relationships that could be construed as a potential conflict of interest.

## Footnotes

1 http://wishart.biology.ualberta.ca/cgview/

## Supplementary Material

Supplementary Figure 1 Biofilm removal activity of antibiotics. The reduction of biofilm after two antibiotics treatment with different concentrations (MIC, 2 × MIC, 4 × MIC, 8 × MIC, 16 × MIC, 32 × MIC) for 6 h. Biofilm formation state was indicated by OD_590_ values. **(A)** Different concentrations of tetracycline to remove biofilm. **(B)** Different concentrations of cefoperazone sodium to remove biofilm. ^∗∗^*P* values of <0.05 compared with the PBS treated control. The mentioned experiment was repeated three times. Each data is expressed as mean ± SD from three biological experiments.
