# Supplementary figures and images for: Genomic, Morphological and Functional Characterization of Virulent Bacteriophage IME-JL8 Targeting Citrobacter freundii
Source: Front Microbiol. 2020 Nov 19;11:585261. doi: 10.3389/fmicb.2020.585261 (PMC7717962; doi:10.3389/fmicb.2020.585261)

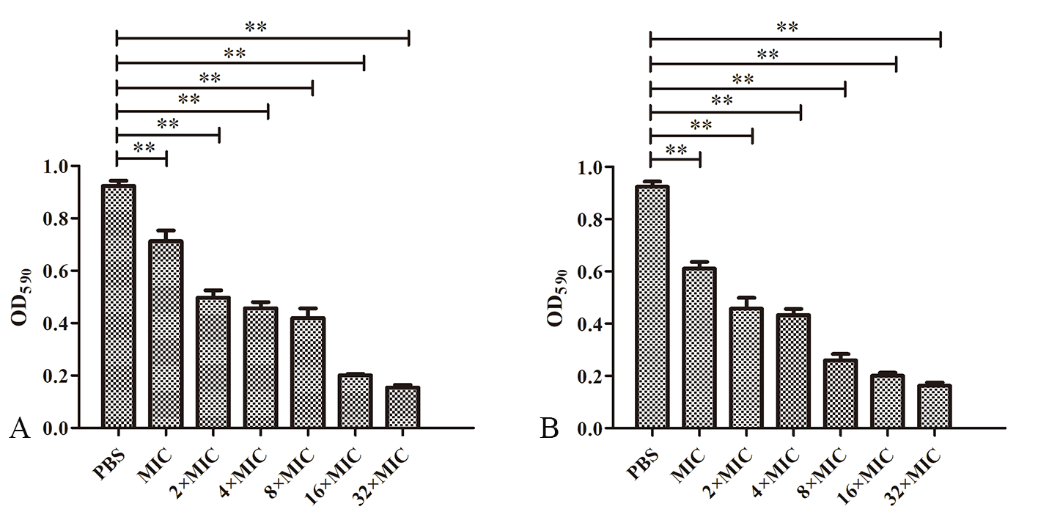

Supplement: Supplementary Figure 1 — Biofilm removal activity of antibiotics. The reduction of biofilm after two antibiotics treatment with different concentrations (MIC, 2 × MIC, 4 × MIC, 8 × MIC, 16 × MIC, 32 × MIC) for 6 h. Biofilm formation state was indicated by OD590 values. (A) Different concentrations of tetracycline to remove biofilm. (B) Different concentrations of cefoperazone sodium to remove biofilm. ∗∗P values of <0.05 compared with the PBS treated control. The mentioned experiment was repeated three times. Each data is expressed as mean ± SD from three biological experiments. [file Data_Sheet_1.docx]
