# Supplementary material for: Genomic, Morphological and Functional Characterization of Virulent Bacteriophage IME-JL8 Targeting Citrobacter freundii
Source: Front Microbiol. 2020 Nov 19;11:585261. doi: 10.3389/fmicb.2020.585261 (PMC7717962; doi:10.3389/fmicb.2020.585261)
Supplement: Supplementary file 2 [file Table_1.docx]

Table.S1 Results of drug sensitivity of CF8

| Antibiotics | Concentration | Diameters of | Sensitivity |
| --- | --- | --- | --- |
|  | (μg/disc) | inhibition (mm) |  |
| Penicillin | 10 U | 13 | I |
| Oxacillin | 1 | 0 | R |
| Ampicillin | 10 | 11 | I |
| Carbenicillin | 100 | 14 | I |
| Piperacillin | 100 | 14 | I |
| Cefalexin | 30 | 0 | R |
| Minocycline | 30 | 10 | I |
| Compound Sulfamethoxazole | 23.75/1.25μg / disc | 0 | R |
| Doxycycline | 30 | 8 | R |
| Ceftazidime Pentahydrate | 30 | 0 | R |
| Ceftriaxone Sodium | 30 | 22 | S |
| Cefoperazone sodium | 75 | 23 | S |
| Amikacin | 30 | 14 | I |
| Tetracycline | 30 | 16 | S |
| Clindamycin | 2 | 0 | R |
| Kanamycin | 30 | 9 | R |
| Furazolidone | 300 | 12 | I |
| Gentamicin | 10 | 12 | I |
| Neomycin | 30 | 12 | I |
| Cefuroxim | 30 | 0 | R |
| Midecamycin | 30 | 0 | R |
| Norfloxacin | 10 | 7 | R |
| Ofloxacin | 5 | 11 | I |
| Ciprofloxacin | 5 | 9 | R |
| Vancomycin | 30 | 12 | I |
| Polymyxin B | 300 IU | 10 | I |
| Cefradine | 30 | 0 | R |
| Chloramphenicol | 30 | 19 | S |
| Erythromycin | 15 | 0 | R |
| Cefamedin | 30 | 0 | R |

Note: S: sensitivity (diameter >15 mm); I: intermediate sensitive (15 mm ≥ diameter ≥10 mm); R: resistant; (10 mm>diameter ≥0 mm).
